# Bicuspid Aortic Valve Associated with Severe Aortic Regurgitation and Large Aortic Root Aneurysm

**DOI:** 10.7759/cureus.7321

**Published:** 2020-03-18

**Authors:** Alejandro Sanchez-Nadales, Miguel Treminio Quezada, Valentina Celis, Jessica Navarro

**Affiliations:** 1 Internal Medicine, Advocate Illinois Masonic Medical Center, Chicago, USA; 2 Internal Medicine, Mount Sinai Medical Center, Miami, USA

**Keywords:** echocardiogram, cta, aortic regurgitation, bicuspid aortic valve, aortic aneurysm, bentall procedure

## Abstract

We described the case of a 30-year-old male who came to the Emergency Department complaining of left shoulder pain and dyspnea under exertion. A bicuspid aortic valve and aneurysm of root and ascending aorta were diagnosed. These were initially managed with medical therapy and ultimately with definitive surgical correction.

## Introduction

Bicuspid aortic valve (BAV) is the most common congenital heart defect; it has a prevalence of approximately 1% and it is more common in men [1]. BAV is a clinically relevant entity, usually associated with valve-related complications as infective endocarditis and vascular anomalies such as aortic dilatation, which is the reason why these patients have a higher risk of requiring surgery for valvular or aortic disease [2]. We present the case of a 30-year-old male with a bicuspid aortic valve with aortic root and ascending aorta aneurysm. 

## Case presentation

**Figure 1:**
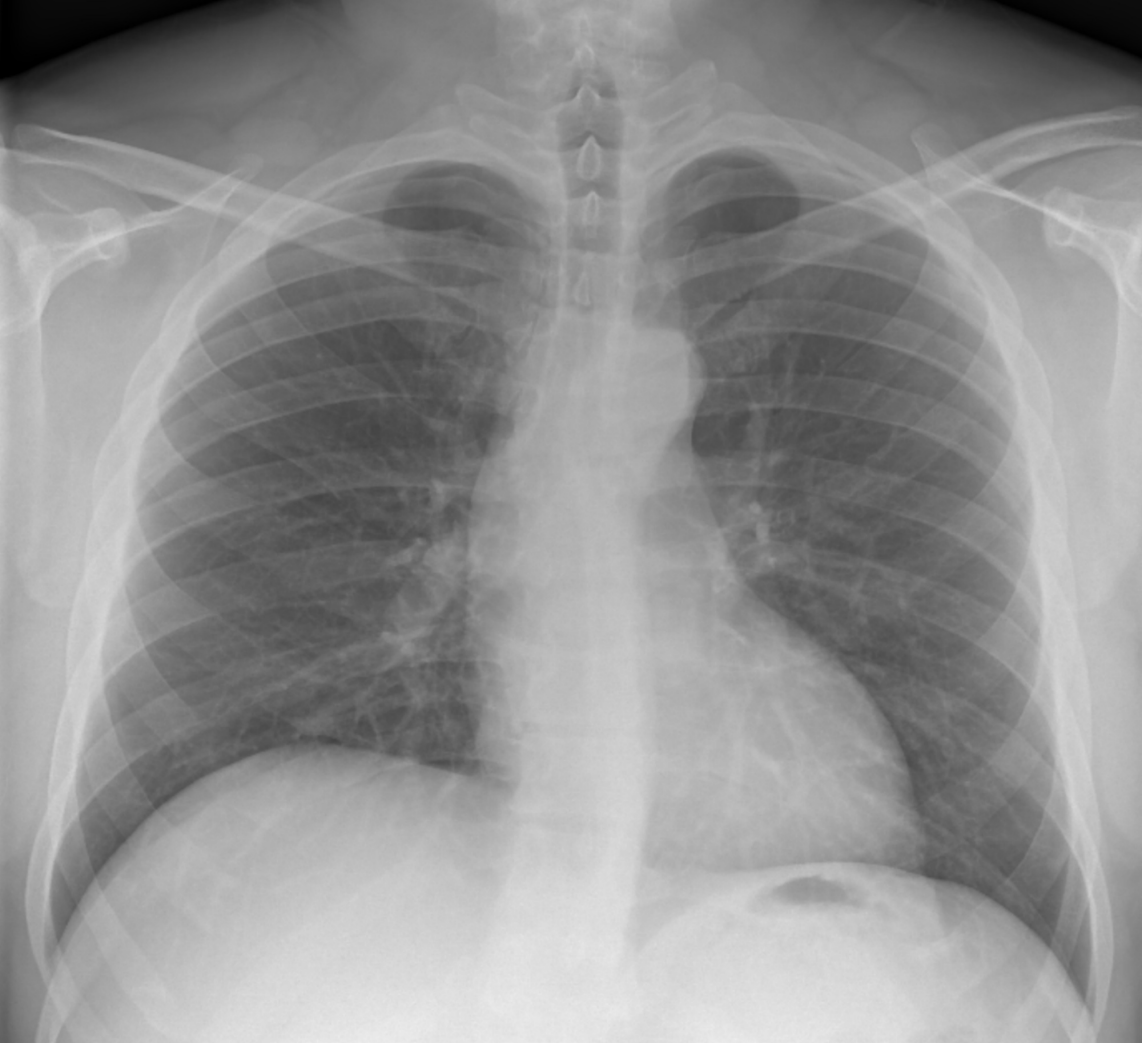
Chest X-Ray (PA) Unremarkable Chest X-Ray. Regular heart silhouette size and clear lung fields without pleura effusion, pulmonary edema, or vasculature congestion.

**Figure 2:**
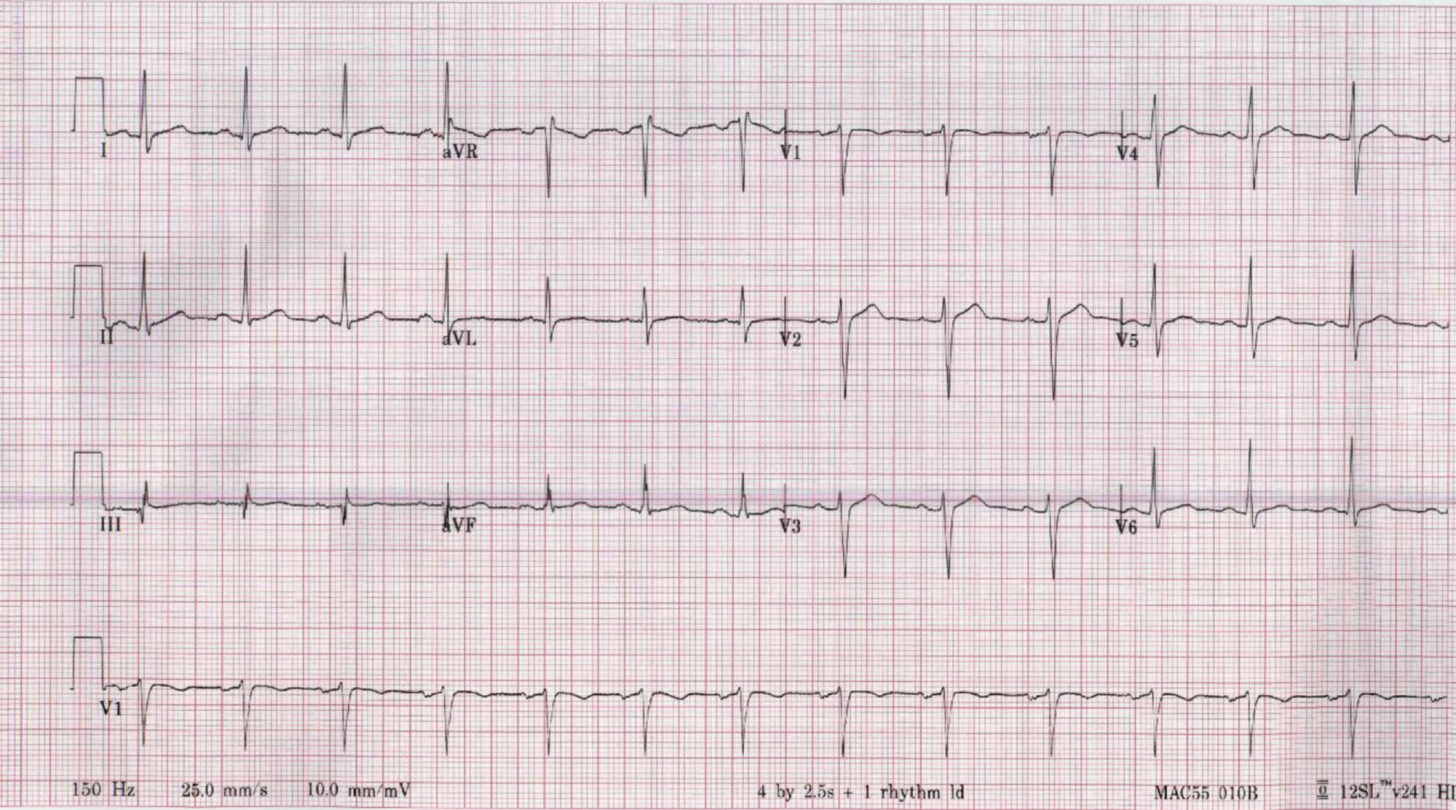
12 Lead Electrocardiogram. Normal sinus rhythm, normal axis, no findings of chamber enlargements, no ST segment, or T wave changes.

A 30-year-old male with a past medical history of asthma and gastroesophageal reflux disease presented with intermittent sharp left shoulder pain and radiated to the neck and arms for the last two weeks, in addition to progressive dyspnea on exertion for the past couple of months. He was previously able to walk and run long distances without symptoms but showed profound shortness of breath with minimal exertion associated with palpitations at the time of presentation. He denied lifting heavy objects or recent trauma, syncopal events, lightheadedness or dizziness. Family history was pertinent to premature coronary artery disease. He denied consumption of tobacco, alcohol, and illicit drug use. Physical examination revealed bilateral lung base crackles and trace pedal edema. Chest X-ray was grossly normal (Figure 1), and had a normal sinus rhythm with inadequate R-wave progression at the electrocardiogram (Figure 2).

Trans-esophageal echocardiogram (TEE) and CT angiography (CTA) scan were ordered owing to findings at 2D Trans-thoracic Echocardiogram (TTE), which revealed left ventricle ejection fraction at 50%, grade 1 diastolic dysfunction, moderate to severe aortic regurgitation and dilated aorta. TEE proved a bicuspid aortic valve with normal cusp separation with moderate eccentric regurgitation, and confirmed the presence of a dilated aortic root measuring 4.8 cm and dilated ascending aorta measuring 4.2 cm (Videos 1, 2).

**Video 1:** Initial Trans-esophageal Echocardiogram. Bicuspid valve anatomy with normal cusp separation.

**Video 2:** Initial Trans-esophageal Echocardiogram. The aortic root is dilated (measuring 4.8 cm), the ascending aorta is dilated (measuring 4.2 cm). Mild to moderate eccentric aortic valve regurgitation.

**Figure 3:**
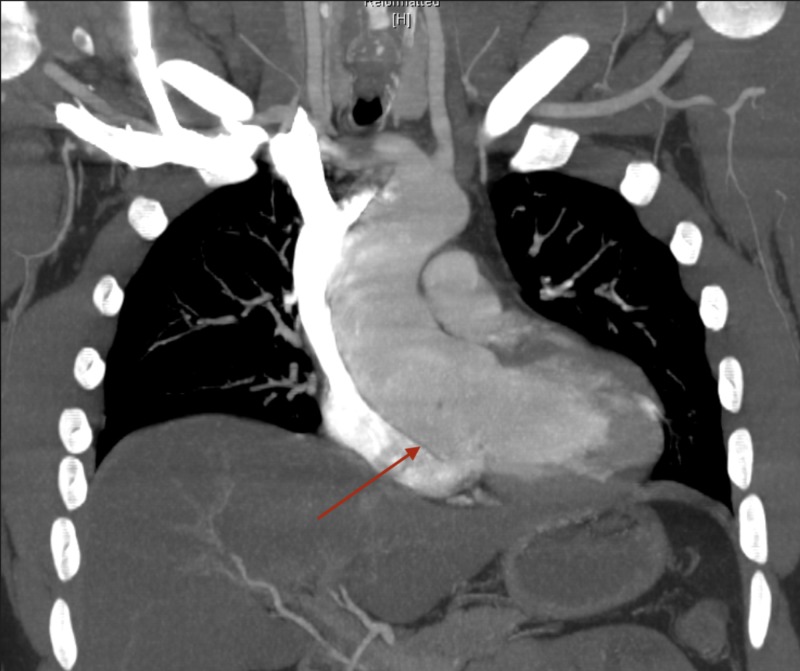
Computerized tomography angiography (CTA) of the chest. Dilatation of the ascending thoracic aorta (from the sinuses of Valsalva to the proximal aortic arch). Cross-sectional aortic measurements are as below: Sinuses of Valsalva: 6.0 x 4.5 cm. Sinotubular junction: 4.7 cm x 4.7 cm. Ascending aorta: 4.7 x 4.5 cm.

**Figure 4:**
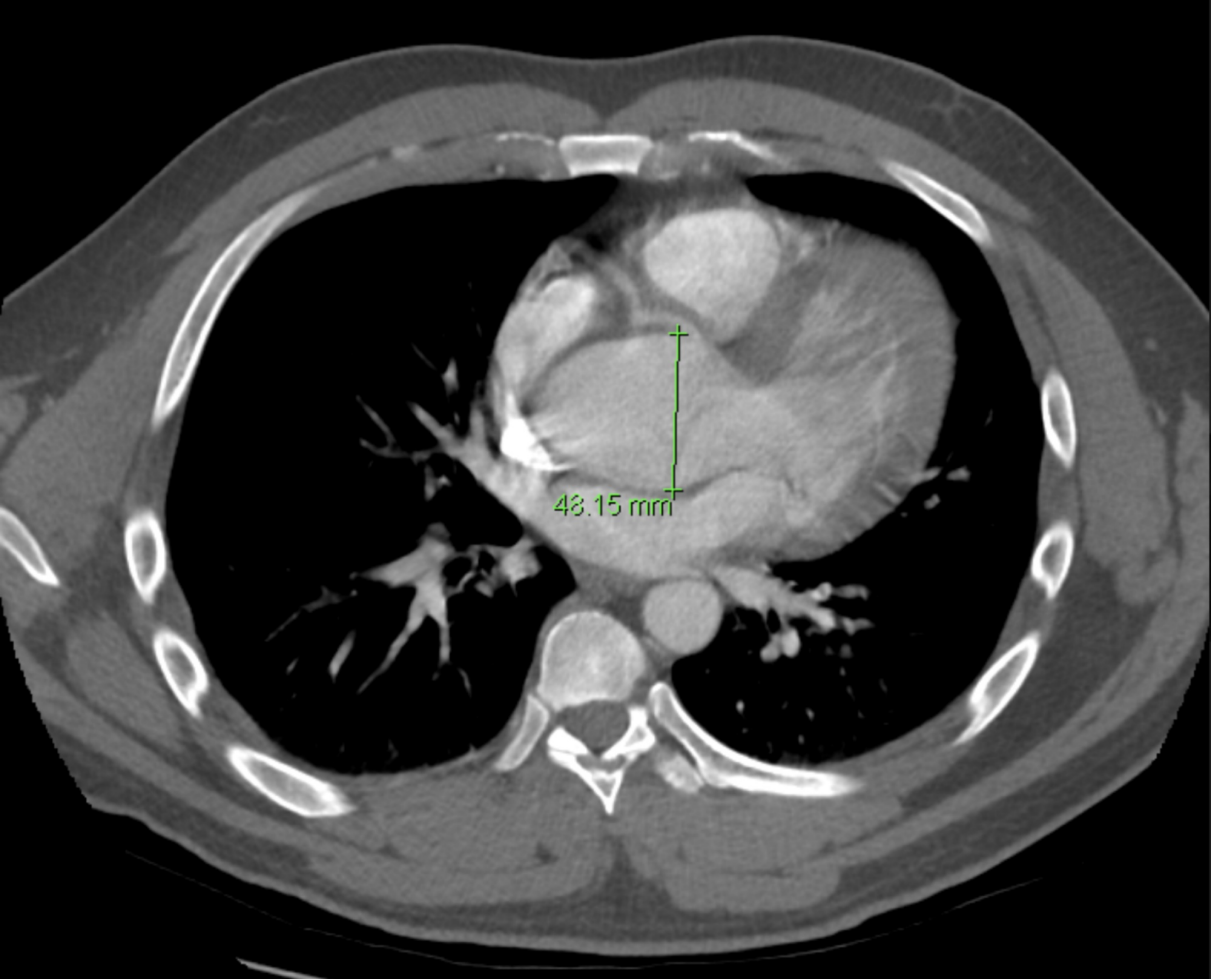
Computerized tomography angiography (CTA) of the chest. Dilatation of the ascending thoracic aorta (from the sinuses of Valsalva to the proximal aortic arch). Cross-sectional aortic measurements are as below: Sinuses of Valsalva: 6.0 x 4.5 cm. Sinotubular junction: 4.7 cm x 4.7 cm. Ascending aorta: 4.7 x 4.5 cm.

**Figure 5:**
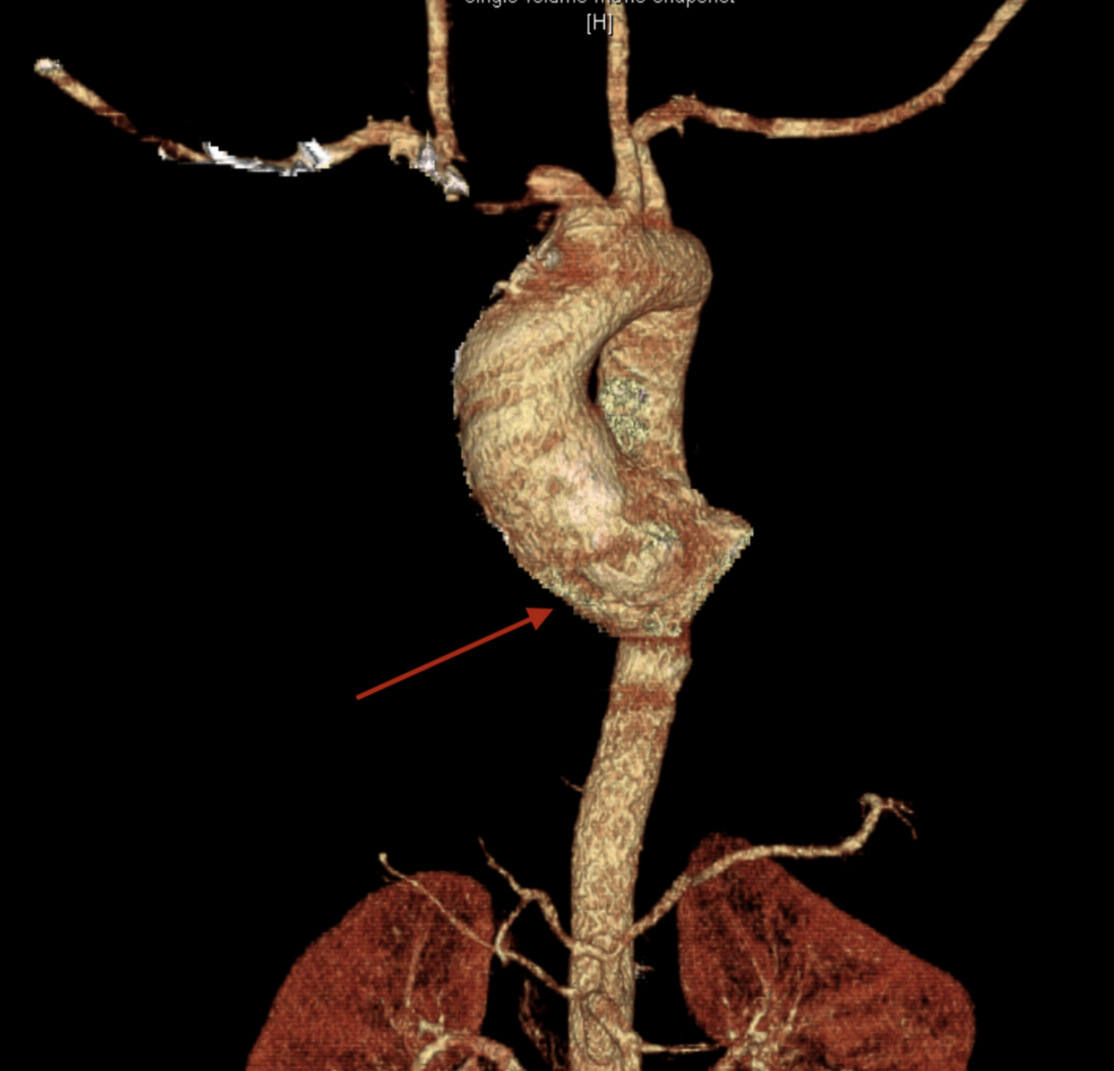
Visual Three-Dimensional Reconstruction of the Aorta.

Chest CTA reported no evidence of aortic dissection, rupture, or intramural hematoma, with aneurysm of sinuses of Valsalva (Figures 3-5).

**Figure 6:**
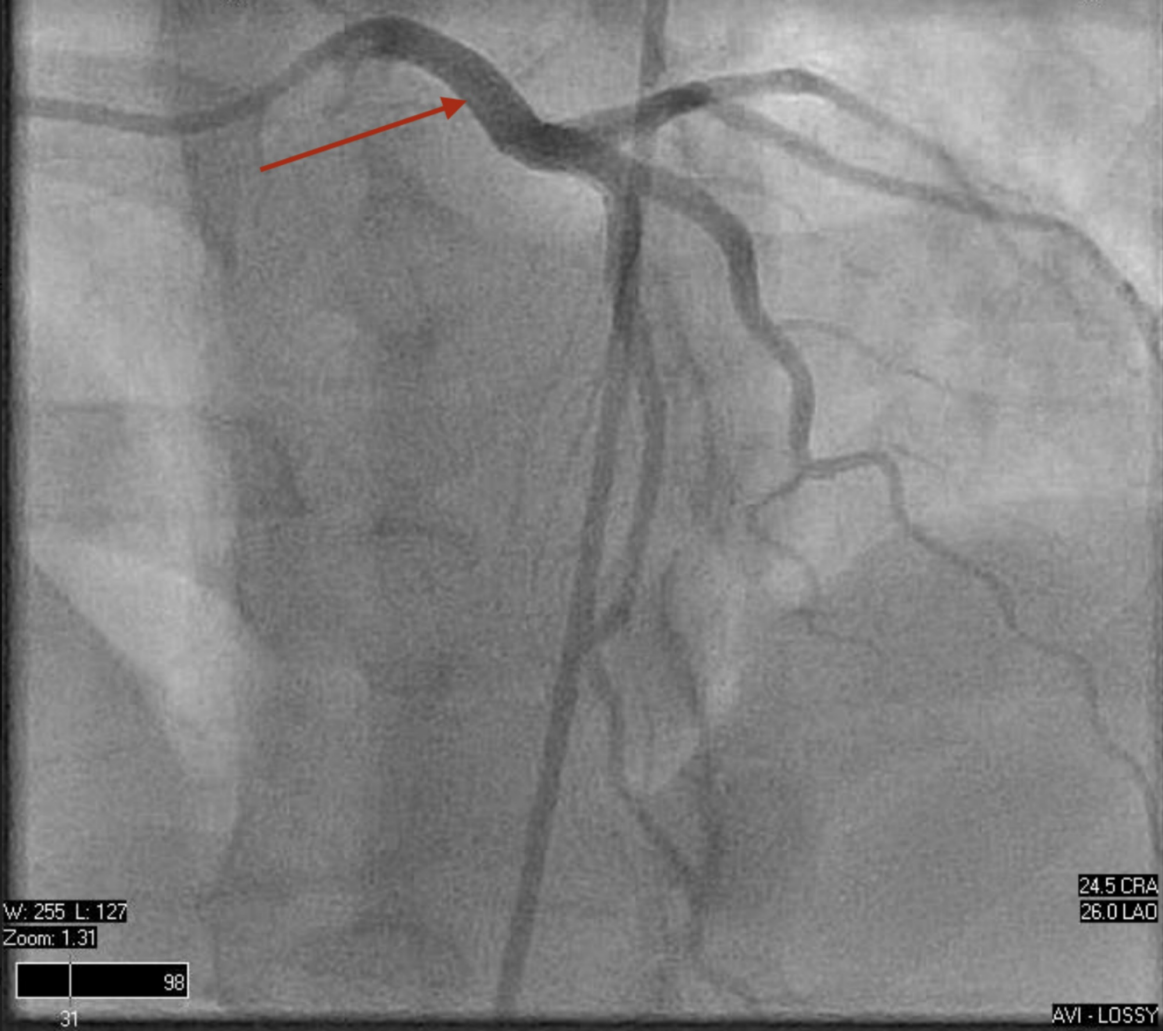
Left heart catheterization. Normal coronary anatomy including the left main coronary artery, left anterior descending artery, and left circumflex.

**Figure 7:**
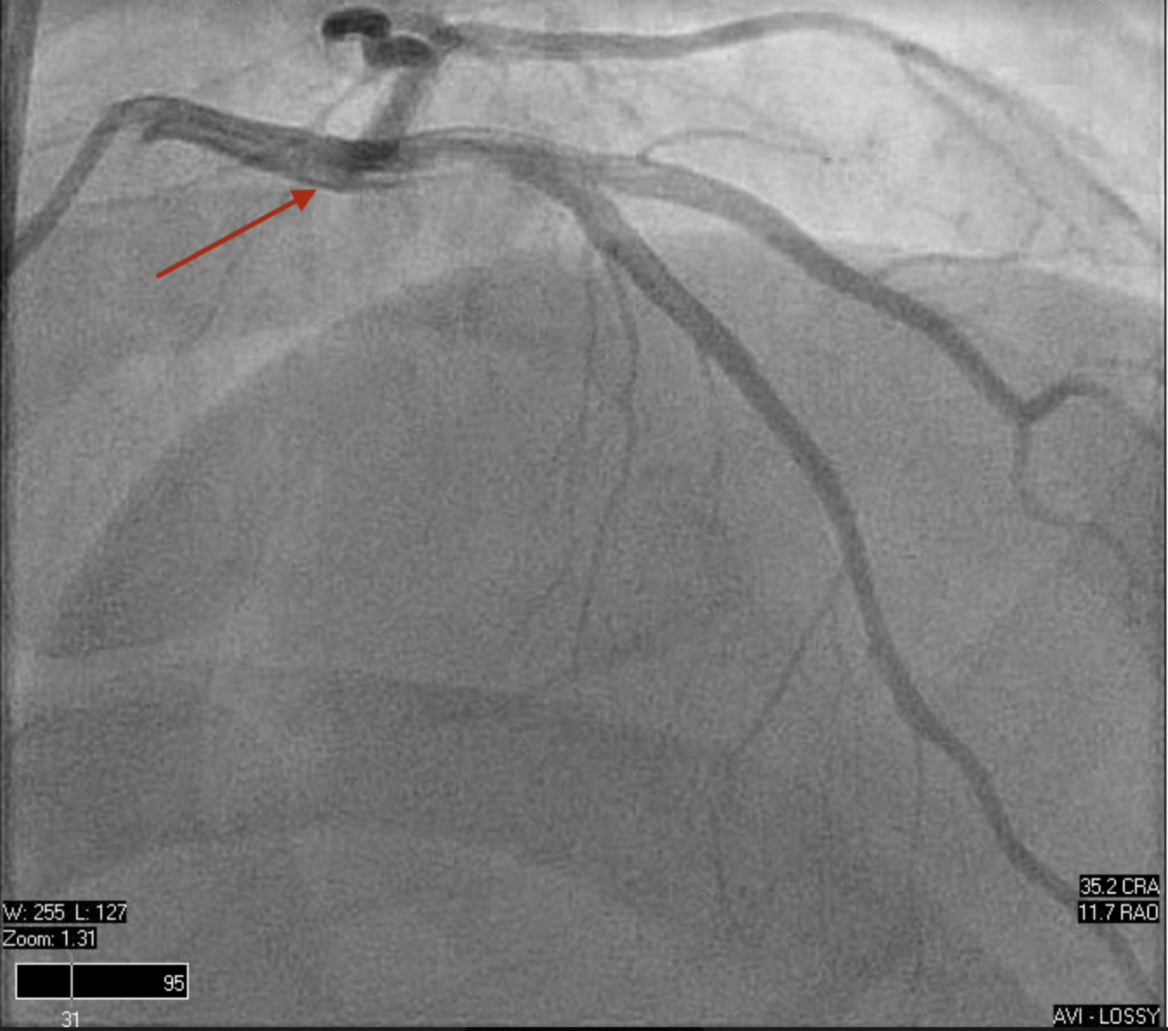
Left heart catheterization. Normal coronary anatomy including the left main coronary artery, left anterior descending artery, and left circumflex.

**Figure 8:**
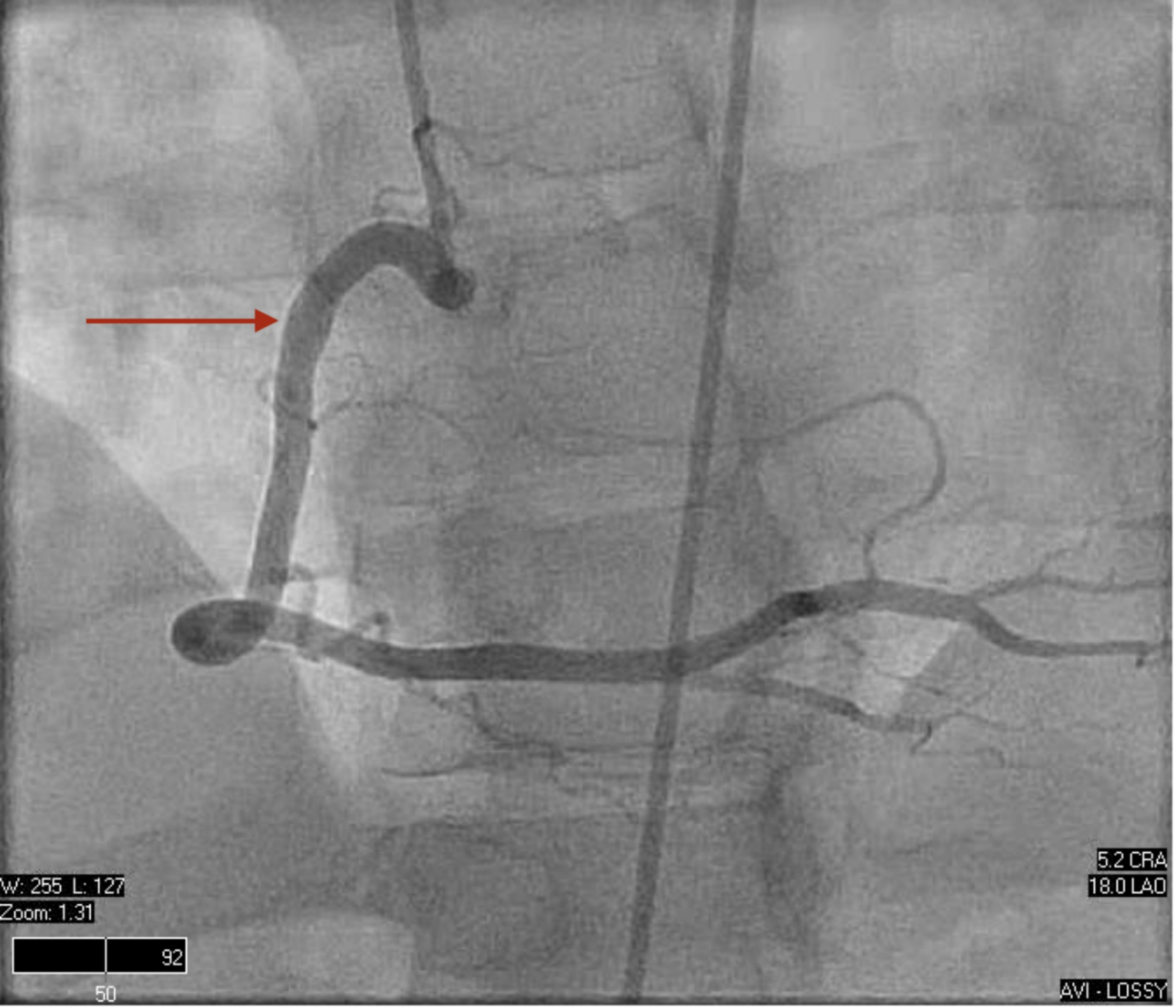
Right heart catheterization. Normal coronary anatomy including the right coronary artery.

Left and right heart catheterization were performed, proving normal hemodynamic parameters and unremarkable coronary anatomy (Figures 6-8).

Cardiothoracic surgery service was consulted, and it was decided to address the management with a Bentall surgical correction. After explaining the methods, benefits, and risks involved, the patient and family agreed with surgical intervention. A St. Jude valve conduit was placed without acute complications during the procedure, and close monitoring took placed in the surgical intensive care unit for the next five days. The patient was discharged after an appropriate recovery time on temporary anticoagulation with a vitamin K antagonist. The patient exhibited significant improvement in symptoms and functional capacity as an outpatient follow up. A follow-up TTE was performed eight months after discharge, showing a mechanical aortic valve functioning normally, without stenosis or regurgitation and normal cusp separation (Video 3).

**Video 3:** Outpatient follow-up Trans-thoracic Echocardiogram (Parasternal long-axis view). Normal systolic function with estimated LVEF at 60-65%. Mechanical aortic valve with normal function (No stenosis or regurgitation).

## Discussion

BAV increased the risk for aortic root dilatation; the etiology behind this association is congenital as both structures have a common embryological origin as they originate from the neural crest cells [3]. An aortic aneurysm is associated with genetic disorders that cause medial degeneration like a bicuspid aortic valve. Risk factors for an aneurysm in patients with BAV such as tobacco abuse and hypertension should be aggressively addressed [4]. Patients with an aortic aneurysm are habitually asymptomatic, but when symptoms are present, they usually manifest as chest and back discomfort. The initial diagnosis of aortic aneurysms is made with TTE, which is indicated to assess the aortic root and proximal ascending aorta [5]. Computed tomography or magnetic resonance imaging can help to visualize the entire aorta and determine the extent of the disease [6]. Medical treatment includes beta-adrenergic blockers, angiotensin-converting-enzyme inhibitors, and angiotensin-receptor blockers [5]. For asymptomatic patients, elective surgical repair is indicated for rapidly growing aneurysms. Aneurysm size thresholds for elective surgery differ based on etiology and anatomic area and are typically lower in patients with genetic syndromes [7]. The Bentall procedure is a type of cardiac surgery involving a composite graft replacement of the aortic valve, aortic root, and ascending aorta, with re-implantation of the coronary arteries into the graft. 

## Conclusions

BAV is a clinically relevant entity, usually associated with valve-related complications such as infective endocarditis and vascular anomalies such as aortic dilatation. For patients who are known or discovered to have BAV, it is crucial to do a thoughtful evaluation looking for complications. One of the most common complications is the formation of an aortic aneurysm. Patients usually present asymptomatic or can develop symptoms as presented in our case. Imaging and hemodynamic assessment are essential to determine the appropriate treatment. Surgical treatment with the Bentall procedure represents the gold standard intervention in patients with aneurysm disease involving the aortic root.
